# Oncology patient-reported claims: maximising the chance for success

**DOI:** 10.3332/ecancer.2011.212

**Published:** 2011-05-09

**Authors:** H Kitchen, D Rofail, M Caron, M-P Emery

**Affiliations:** 1 Mapi Values, Cheshire, UK; 2 Mapi Research Trust, Lyon, France

## Abstract

**Objectives/purpose:**

To review Patient Reported Outcome (PRO) labelling claims achieved in oncology in Europe and in the United States and consider the benefits, and challenges faced.

**Methods:**

PROLabels database was searched to identify oncology products with PRO labelling approved in Europe since 1995 or in the United States since 1998. The US Food and Drug Administration (FDA) and the European Medicines Agency (EMA) websites and guidance documents were reviewed. PUBMED was searched for articles on PRO claims in oncology.

**Results:**

Among all oncology products approved, 22 were identified with PRO claims; 10 in the United States, 7 in Europe, and 5 in both. The language used in the labelling was limited to benefit (e.g. “…resulted in symptom benefits by significantly prolonging time to deterioration in cough, dyspnoea, and pain, versus placebo”) and equivalence (e.g. “no statistical differences were observed between treatment groups for global QoL”). Seven products used a validated HRQoL tool; two used symptom tools; two used both; seven used single-item symptom measures (one was unknown). The following emerged as likely reasons for success: ensuring systematic PRO data collection; clear rationale for pre-specified endpoints; adequately powered trials to detect differences and clinically significant changes; adjusting for multiplicity; developing an *a priori* statistical analysis plan including primary and subgroup analyses, dealing with missing data, pooling multiple-site data; establishing clinical versus statistical significance; interpreting failure to detect change. End-stage patient drop-out rates and cessation of trials due to exceptional therapeutic benefit pose significant challenges to demonstrating treatment PRO improvement.

**Conclusions:**

PRO labelling claims demonstrate treatment impact and the trade-off between efficacy and side effects ultimately facilitating product differentiation. Reliable and valid instruments specific to the desired language, claim, and target population are required. Practical considerations include rationale for study endpoints, transparency in assumptions, and attention to subtle variations in data.

## Background

Patient Reported Outcome (PRO) questionnaires are useful in assessing any aspect of patients’ health status (disease and care) from their perspective [1]. They cover single and multi-dimensional concepts including, but not limited to, symptoms (e.g. nausea, dyspnoea), functioning, health-related quality of life (HRQoL), treatment satisfaction, and adherence [2]. Essentially, PROs provide an important perspective on how patients feel and function that cannot usually be adequately captured by clinical measures [3].

Although there are some evidence to suggest a relationship between tumour response and PRO data [4] traditionally, oncology clinical trial endpoints have focused on clinical measures such as tumour response rate and time to tumour progression (TTP) [5]. Nevertheless, improvement in long-term survival rates in some cancers, such as testicular [6] and recurrent breast cancer [7], have increased the need for long-term treatments for which impact upon patient symptoms and HRQoL is a key concern.

Recently, regulatory agencies, the US Food and Drug Administration (FDA) and European Medicines Agency (EMA), emphasised the importance of PRO data and issued guidance on the incorporation of PRO instruments into clinical trials [1,8]. Including PRO endpoints in phase II and III clinical trials for oncology products can demonstrate treatment impact from the patient’s perspective and make substantial contributions to the awareness of the disease burden on patients. PRO data can also provide significant prognostic information beyond standard clinical measure [9–11].

## Methods

The PROLabels database was searched to identify oncology products with PRO labelling which had been approved in Europe since 1995 or in the United States since 1998. The FDA and EMA websites and guidance documents were reviewed. In addition, PUBMED was searched for relevant articles using the following keywords: ‘oncology’ OR ‘cancer’ AND ‘patient-reported outcomes’ AND ‘claim’, ‘label’ or ‘regulatory’.

Data were extrapolated from the Drug Product Label and the Summary Base Approval (SBA) (as available) including: the brand and generic product name, date of approval, label indication, type of PRO claim (e.g. HRQoL, symptoms, functioning), PRO claim language, PRO instruments used, and PRO endpoint status. The PRO instruments used to obtain labelling claims were reviewed in light of FDA PRO guidance [12].

## Results

### PRO label claims granted in oncology products by FDA and EMA

A review of the PROLabels website (http://www.mapi-prolabels.org) revealed over 125 oncology products that were approved by FDA and/or EMA between 1995 and 2009. Of these, 22 were identified with PRO claims; 10 in the United States, 7 in Europe, and 5 in both (Table 1).

The majority of the label claims approved by both the FDA and EMA were based on signs and symptoms: six products obtained a label claim for pain, six products obtained a label claim for emesis and five products obtained a label claim for nausea. The language used in label claims was limited to showing benefit and equivalence (Table 2)

### PRO questionnaires used to support label claims in oncology products

Looking at the label claims that have been granted after FDA draft guidance in 2006 (Table 3), a diverse range of instruments have been utilised to assess the impact of treatment upon patient symptoms and aspects of functioning, from single-item visual analogue scales to multidimensional questionnaires such as the Functional Assessment of Cancer Therapy–Lung (FACT-L).

A review of the PROs used to obtain label claims in oncology products revealed that, in light of FDA guidance, they are generally not ‘fit for purpose’ i.e. the majority of the instruments were developed without input from the intended treatment population.

## Discussion

### Challenges in obtaining PRO label claims in oncology

There are a number of potential challenges involved in achieving a PRO label claim for oncology products. First, the FDA guidance states *“Generally, findings measured by a well-defined and reliable PRO instrument in appropriately designed investigations can be used to support a claim in medical product labelling if the claim is consistent with the instrument’s documented measurement capabilities”* [12]. This requires content validity to be established, including qualitative research, to ensure that items measure the intended concept, are relevant, comprehensive and understood by the intended population. PRO instruments should also be reliable, responsive, and able to detect change in the intended treatment population.

**Figure 1:**
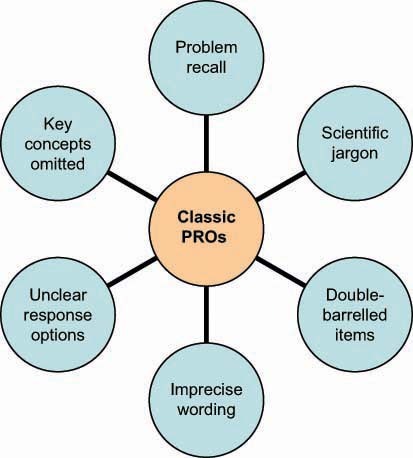
Common problems with classic PRO measurements.

Figure 1 shows further common problems with ‘classic’ PRO instruments including inappropriate recall period and imprecise wording which can be problematic for patients to complete, therefore threatening the consistency and validity of data obtained.

Furthermore, there are specific challenges associated with oncology clinical trials, in particular the complexity of demonstrating symptom or HRQoL improvement in an indication where patients get progressively worse. Demonstrating long-term impacts of treatments requires the sample to be retained for the duration of the trial. This may be especially difficult in samples of late-stage and end-stage patients. In addition, oncology trials are often un-blinded which poses a problem for the accuracy of PRO data which is frequently subject to selection bias and reporting bias [13].

### How to maximise chances for success in achieving a PRO label claim

The following may contribute towards obtaining an oncology PRO label claim:

- ensuring systematic PRO data collection and clear rationale for pre-specified endpoints
- establishing sufficient sample size and adequately powered trials to detect differences and clinically significant changes, adjusting for multiplicity
- developing an *a priori* statistical analysis plan including primary and subgroup analyses
- establishing ‘stringent’ missing data criteria, adjusting for multiple comparisons, and being sensitive to pooling multiple-site data
- establishing clinical versus statistical significance with clear *a priori* responder definitions
- interpreting failure to detect change

**Figure 2:**
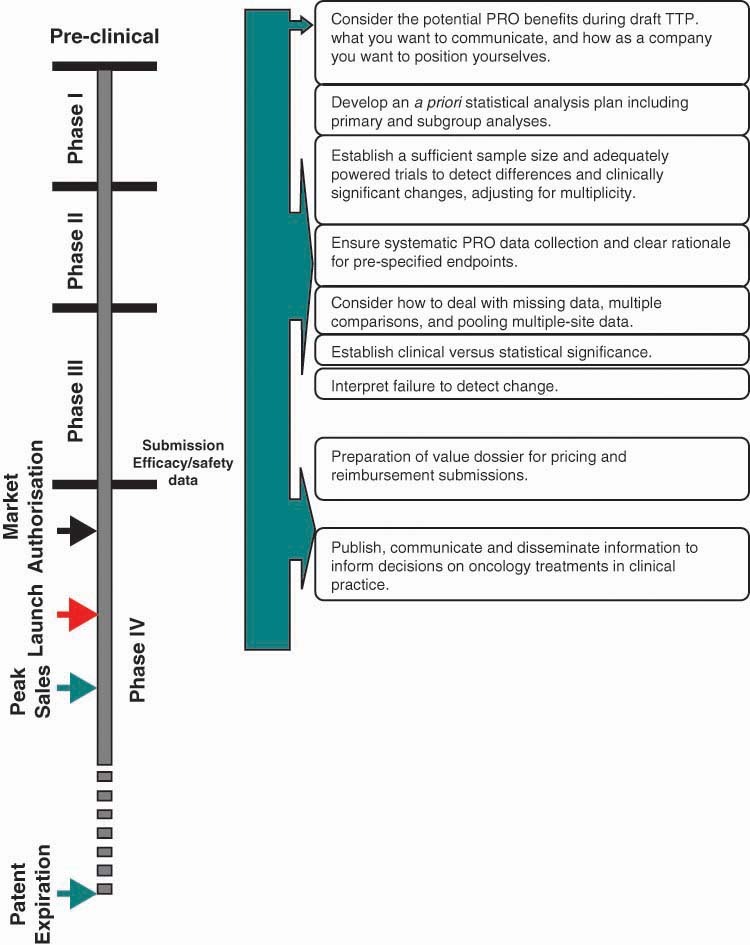
Strategic considerations across an anti-cancer product life-cycle.

These considerations should be taken at various points during the product life-cycle. Figure 2 provides an overview of when consideration of these factors during the product lifecycle is recommended. However, early planning to select, develop, and validate a PRO instrument is advisable in order to overcome these challenges and obtain a PRO label claim for an oncology product.

### Study limitations and future recommendations

This review focussed on the use of PROs in clinical trials where a label claim has been granted. It is important to recognise the limitations that this presents. Due to availability of information in the public domain, it was not possible to assess oncology studies where PRO instruments have been used inappropriately or unsuccessfully. Future research on a study-by-study basis would provide further insight into the successful use of PROs in oncology clinical trials.

This review indicates that PRO instruments can be used to demonstrate the impact of new treatments on symptoms, side effects, functioning, and aspects of overall HRQoL. When incorporated into clinical trials PRO instruments can provide an important assessment of treatment benefit to patients. Further research with patients in disease-specific populations would be useful to investigate key concepts important to patients in order to identify appropriate PRO instruments.

## Conclusions

PRO labelling claims demonstrate treatment impact, and the trade-off between efficacy and side effects ultimately facilitating product differentiation. Reliable and valid instruments specific to the desired language, claim, and target population are required. Practical considerations include rationale for study endpoints, transparency in assumptions, and attention to subtle variations in data.

## Figures and Tables

**Table 1:**
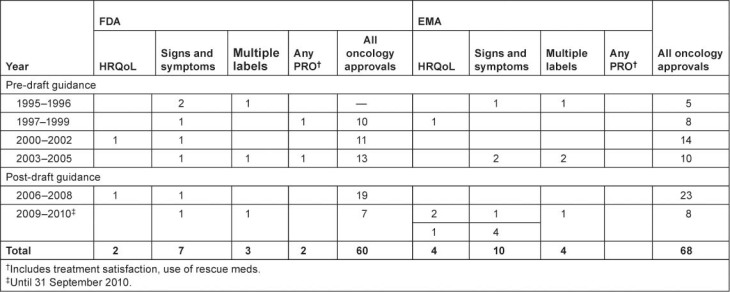
PRO label claims granted in oncology products by FDA and EMA, 1995–2010

**Table 2:**
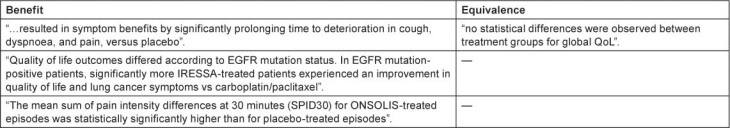
Language used in PRO label claims

**Table 3:**
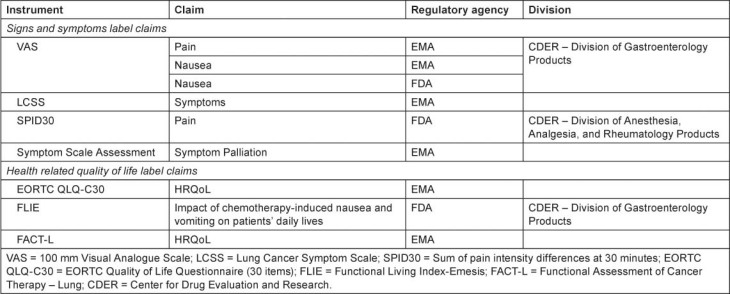
PRO instruments used to support label claims, post 2006
